# Construction and screening of a glycosylphosphatidylinositol protein deletion library in *Pichia pastoris*

**DOI:** 10.1186/s12866-020-01928-y

**Published:** 2020-08-24

**Authors:** Pan Wang, Ying Lin, Chengjuan Zou, Fengguang Zhao, Shuli Liang, Suiping Zheng, Shuangyan Han

**Affiliations:** Guangdong Key Laboratory of Fermentation and Enzyme Engineering, School of Biology and Biological Engineering, South China University of Technology, Guangzhou, 510006 Guangdong China

**Keywords:** GPI protein, Deletion, Phenotypic screen, *Pichia pastoris*

## Abstract

**Background:**

Glycosylphosphatidylinositol (GPI)-anchored glycoproteins have diverse intrinsic functions in yeasts, and they also have different uses in vitro. In this study, the functions of potential GPI proteins in *Pichia pastoris* were explored by gene knockout approaches.

**Results:**

Through an extensive knockout of GPI proteins in *P. pastoris*, a single-gene deletion library was constructed for 45 predicted GPI proteins. The knockout of proteins may lead to the activation of a cellular response named the ‘compensatory mechanism’, which is characterized by changes in the content and relationship between cell wall polysaccharides and surface proteins. Among the 45 deletion strains, five showed obvious methanol tolerance, four owned high content of cell wall polysaccharides, and four had a high surface hydrophobicity. Some advantages of these strains as production hosts were revealed. Furthermore, the deletion strains with high surface hydrophobicity were used as hosts to display *Candida antarctica* lipase B (CALB). The strain gcw22Δ/CALB-GCW61 showed excellent fermentation characteristics, including a faster growth rate and higher hydrolytic activity.

**Conclusions:**

This GPI deletion library has some potential applications for production strains and offers a valuable resource for studying the precise functions of GPI proteins, especially their putative functions.

## Background

Glycosylphosphatidylinositol (GPI)-anchored proteins are found in all eukaryotic cells. They harbor GPI-anchoring machinery and utilize the anchor to express proteins on the cell surface. Precursors of GPI anchored proteins contain an N-terminal signal sequence for import into the ER and a C-terminal signal for GPI anchoring [1]. In yeast, the GPI anchor is essential for viability and maintenance of normal cell morphology [2, 3]. These GPI-cell wall proteins (CWPs) can be grouped into different classes based on their functions. Some GPI proteins play a structural role and may provide stretch resistance by interacting with glucans and other wall components or by interacting with each other through noncovalent bonds and disulfide bridges. Other GPI proteins may act as enzymes that make and break glycosidic linkages, and the rest are required for elaboration of the cell wall and its reshaping during bud emergence, cell separation, mating or entry into stationary phase.

Yeast cells elicit a rescue mechanism called the ‘compensatory salvage response’, which provides compensatory synthesis of cell wall material and changes the cross-linking type between cell wall polymers necessary for maintenance of cellular integrity and yeast survival [4, 5]. Many responses occur under the control of the cell wall integrity signal transduction pathway and high osmotic glycerol pathway, which transmit wall stress signals from the cell surface, activate the Slt2 MAP kinase, and regulate the production and polarized delivery of various components to the site of cell wall remodeling. In particular, the cell wall structure needs to be remodeled under those stress conditions with a direct impact on cell wall integrity [6]. GPI proteins are also essential for maintaining cellular integrity. Quite a few GPI protein deletion strains have been constructed to explore cell properties and protein functions. For example, the loss of Ecm33p affects cell morphology, causes glycosylation defects, results in a short fermentation duration in either a synthetic medium or grape juice, and triggers the activation of the CWI MAPK pathway in *S. cerevisiae* [7, 8]. A strain with knockouts of five GPI proteins (yps1Δ yps2Δ yps3Δ yps6Δ yps7Δ) did not show detectable growth defects under normal growth conditions but appeared to have an efficient ability to prevent the proteolytic degradation of hPTH in fed-batch cultivations compared with other combinations of deletion strains [9]. The stress-induced structural GPI protein SED1 was successfully knocked out in *S. cerevisiae* and used as a host strain. The strain produced an increasing amount of heterologous-displayed enzymes on the yeast cell surface using the SED1 anchoring system [10].

In view of the above phenotypic changes owing to the GPI protein deletion strains, potential strains playing positive roles in the expression of heterologous proteins, changing polysaccharide structure or content, and altering growth mode for survival should be discovered. By a comprehensive genome-wide search, 50 putative GPI-anchored proteins in the *P. pastoris* genome were exploited in previous research, and 16 predicted GPI proteins could act as anchors to successfully display heterologous proteins on the cell surface [11]. This work opened the door to deeply study the role of GPI specifically in *P. pastoris. P. pastoris* is a widely used expression system with a strong promoter alcohol oxidase 1 (AOX1). With this system, recombinant protein expression can be induced 1000-fold upon methanol addition [12, 13]. The display of enzymes on the surface of *P. pastoris* is an active topic in the field of whole-cell biocatalysts. However, the cell surface enzyme activity and protein amount are limited by some factors. For example, the anchorage position of the target protein in the cell wall is an important factor that maximizes the capabilities of engineered yeast cells [14]. Therefore, the functions of GPI-anchored proteins on cell surface display should be further explored.

However, the physiological roles of more than 70% of these predicted GPI-anchored proteins in *P. pastoris* are unknown. In the present study, we hope to obtain more information about the GPI proteins in *P. pasto*ris. The function of GPI proteins in *P. pastoris* could also provide more information on other excellent expression systems [15, 16]. According to the compensatory salvage response, the deletion strains could elicit a series of phenotypic changes, including changes in the cell wall polysaccharide content and protein types. Therefore, a GPI protein deletion library was constructed by knocking out a single predicted GPI protein-encoding gene in *P. pastoris*. Finally, 45 GPI protein deletion strains were successfully obtained, and their characteristics were investigated.

## Results

### Construction of a GPI protein deletion library with the Cre/loxP system

According to the amino acid sequence characteristics of GPI-anchored proteins, Zhang et al. predicted and screened 50 potential GPI-anchored proteins in *P. pastoris* [11]. To explore the functions of these proteins, a GPI protein deletion library with each predicted protein knocked out with the Cre/loxP system was contructed. To maximize the efficiency of gene replacement, the gene knockout expression cassette contained ~ 700 bp homologous flanking regions for every gene. Finally, 17 gene knockout expression cassettes were successfully transformed into GS115. To significantly increase homologous recombination efficiencies in *P. pastoris*, the endogenous gene *ku70* homolog, a key player in nonhomologous-end-joining (NHEJ) repair, was deleted, and the strain GS115 ku70 was constructed [17]. Another 28 gene knockout expression cassettes were transformed into the strain GS115 ku70, and the recombination efficiency significantly improved. Thus, 45 GPI-deficient strains were successfully obtained. The detailed information is shown in Table 1.

**Table 1:** The gene information of GPI proteins in deletion library strains

Strains	Deletion gene			
	Gene name	Size (AA)	NCBI BLAST	Domain description
gcw1Δ	GCW1	538	β-1,3-Glucanosyltransferase	Glucanosyltransferase; X8; Glycoside hydrolase
gcw2Δ	GCW2	535	β-1,3-Glucanosyltransferase	Glucanosyltransferase; X8; Glycoside hydrolase
ku70Δ gcw3Δ	GCW3	307	Hypothetical protein	Superoxide dismutase, Cu/Zn binding domain
gcw4Δ	GCW4	266	Hypothetical protein	No hits found
gcw5Δ	GCW5	203	Hypothetical protein	Flocculin type 3 repeat
gcw6Δ	GCW6	448	Cell wall protein	Glycoside hydrolase, predicted CRH1;Concanavalin A-like lectin/glucanases
gcw7Δ	GCW7	593	Aspartic protease	Peptidase A1; Aspartic peptidase
ku70Δ gcw8Δ	GCW8	448	Putative mannosidase	Glycoside hydrolase; Six-hairpin glycosidase; Mannanendo-1,6-alpha-mannosidase
gcw10Δ	GCW10	251	Hypothetical protein	No hits found
gcw12Δ	GCW12	233	CFEM protein	Extracellular membrane protein, CFEM domain
gcw13Δ	GCW13	294	Hypothetical protein	No hits found
gcw14Δ	GCW14	135	Hypothetical protein	No hits found
gcw15Δ	GCW15	237	Hypothetical protein	No hits found
gcw16Δ	GCW16	1416	Mucin-like protein	Cellulose-binding domain; Uncharacterized domain Flo11-related
gcw17Δ	GCW17	259	Hypothetical protein	No hits found
–	GCW18	1667	Hypothetical protein	Peptidase S8/S53 domain; PT repeat, subtilisin-related
gcw19Δ	GCW19	148	Hypothetical protein	No hits found
gcw21Δ	GCW21	233	Hypothetical protein	No hits found
gcw22Δ	GCW22	839	Hypothetical protein	Uncharacterized domain Flo11-related, N-terminal; Flocculin type 3 repeat
–	GCW23	418	Cell wall protein	Receptor L domain-like
ku70Δ gcw24Δ	GCW24	261	Hypothetical protein	No hits found
ku70Δ gcw25Δ	GCW25	595	Hypothetical protein	Flocculin type 3 repeat
ku70Δ gcw26Δ	GCW26	724	Hypothetical protein	GLEYA adhesin domain
ku70Δ gcw28Δ	GCW28	587	Mucin-like protein	Adhesion domain, bacterial
–	GCW29	1474	Hypothetical protein	PT repeat; Adhesion domain, bacterial
ku70Δ gcw30Δ	GCW30	229	Hypothetical protein	No hits found
ku70Δ gcw31Δ	GCW31	562	Hypothetical protein	Peptidase A1; Aspartic peptidase
ku70Δ gcw32Δ	GCW32	216	Hypothetical protein	No hits found
–	GCW33	527	Aspartic protease	Peptidase A1; Aspartic peptidase
ku70Δ gcw34Δ	GCW34	473	Aspartic protease	Peptidase A1; Aspartic peptidase
ku70Δ gcw35Δ	GCW35	582	Putative aspartic protease	Peptidase A1; Aspartic peptidase
ku70Δ gcw36Δ	GCW36	468	Hypothetical protein	PT repeat
ku70Δ gcw37Δ	GCW37	234	Hypothetical protein	No hits found
ku70Δ gcw39Δ	GCW39	612	Aspartic protease	Peptidase A1; Aspartic peptidase
ku70Δ gcw42Δ	GCW42	248	Hypothetical protein	No hits found
ku70Δ gcw43Δ	GCW43	443	Hypothetical protein	EGF receptor, L domain
ku70Δ gcw45Δ	GCW45	409	Hypothetical protein	PT repeat; Flocculin type 3 repeat
ku70Δ gcw46Δ	GCW46	633	Phospholipase B	Lysophospholipase, catalytic domain; Acyl transferase/acyl hydrolase
ku70Δ gcw48Δ	GCW48	369	Putative protease	SUN Family
ku70Δ gcw49Δ	GCW49	327	Hypothetical protein	No hits found
–	GCW50	454	Hypothetical protein	No hits found
ku70Δ gcw51Δ	GCW51	211	Hypothetical protein	No hits found
ku70Δ gcw52Δ	GCW52	365	Hypothetical protein	No hits found
ku70Δ gcw53Δ	GCW53	354	Hypothetical protein	No hits found
ku70Δ gcw54Δ	GCW54	611	Hypothetical protein	No hits found
ku70Δ gcw56Δ	GCW56	400	Hypothetical protein	No hits found
ku70Δ gcw58Δ	GCW58	194	Hypothetical protein	Stress-induced protein SRP1/TIP1
ku70Δ gcw59Δ	GCW59	469	Putative chitin transglycosidase	Glycoside hydrolase, predicted CRH1;Concanavalin A-like lectin/glucanases
ku70Δ gcw60Δ	GCW60	599	Aspartic protease	Peptidase A1; Aspartic peptidase
gcw61Δ	GCW61	65	Hypothetical protein	No hits found

### Growth of deletion library strains with different carbon sources

**Fig. 1:**
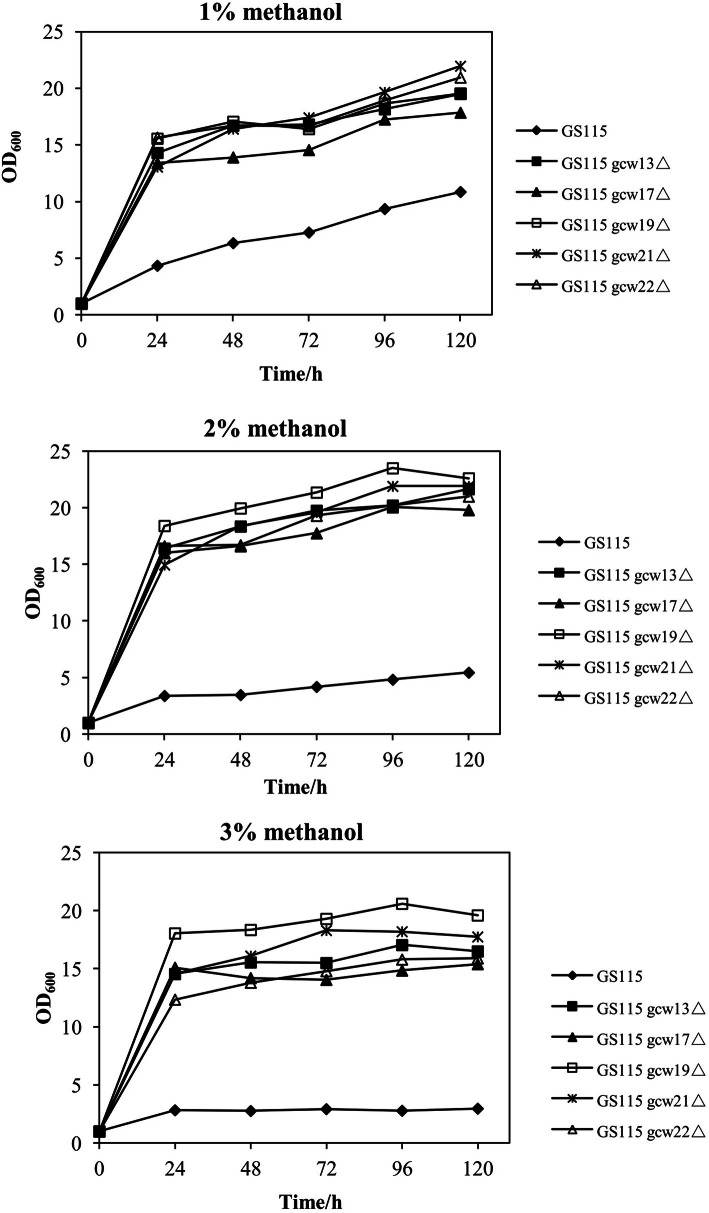
Fermentation curve of deletion strains in media with different methanol concentrations Cells were cultured in BMMY medium for 120 h, and methanol was added every 24 h

Yeast can grow on different carbon sources, which are known to influence their growth behavior. To explore the growth characteristics of the deletion strains, four different carbon source cultures, YPD, YPG, YPM and BMMY, were used. The specific growth rates of all the strains are listed in Table 2. These GPI deletion strains grew normally on YPD and YPG media compared with the control strain, indicating that the knocked out genes are not necessary for yeast cell growth. The utilization rates of different carbon sources showed wide variations. Generally, the cells grew better on the carbon sources with glucose or glycerol but grew poorly with methanol. As a methylotrophic yeast, *P. pastoris* has been given great attention for its ability to utilize low-cost methanol as a sole carbon source to express high levels of recombinant proteins. However, high methanol concentrations often result in severe growth defects, which presents serious issues for industrial applications. Five strains in the GPI deletion library, gcw13Δ, gcw17Δ, gcw19Δ, gcw21Δ, and gcw22Δ, showed obvious variations in the media supplemented with methanol as the carbon source, including the media YPM, 1% BMMY, 2% BMMY and 3% BMMY. Significantly accelerated specific growth rates were observed for the above five strains. Furthermore, the cell growth of these five strains in BMMY medium with shake flasks also exhibited a remarkable growth advantage (Fig. 1), which was similar to the growth state in 96-well plates.

**Table 2:** The specific growth rate μ of deletion library strains

Strains	YPD	YPG	YPM	1%BMMY	2%BMMY	3%BMMY	Strains	YPD	YPG	YPM	1%BMMY	2%BMMY	3%BMMY
GS115	0.288	0.201	0.119	0.129	0.094	0.060	ku70Δ gcw28Δ	0.244	0.165	0.099	0.084	0.063	0.054
gcw1Δ	0.285	0.197	0.110	0.144	0.116	0.076	ku70Δ gcw30Δ	0.271	0.191	0.095	0.103	0.118	0.054
gcw2Δ	0.338	0.243	0.130	0.171	0.140	0.080	ku70Δ gcw31Δ	0.281	0.212	0.096	0.127	0.103	0.053
gcw4Δ	0.271	0.220	0.107	0.156	0.101	0.055	ku70Δ gcw32Δ	0.294	0.216	0.083	0.117	0.099	0.052
gcw5Δ	0.308	0.215	0.098	0.121	0.108	0.067	ku70Δ gcw34Δ	0.301	0.236	0.080	0.131	0.118	0.036
gcw6Δ	0.287	0.208	0.116	0.146	0.099	0.057	ku70Δ gcw35Δ	0.287	0.202	0.086	0.110	0.103	0.045
gcw7Δ	0.287	0.193	0.104	0.106	0.114	0.060	ku70Δ gcw36Δ	0.268	0.182	0.092	0.092	0.069	0.060
gcw10Δ	0.254	0.152	0.120	0.139	0.109	0.054	ku70Δ gcw37Δ	0.315	0.227	0.102	0.129	0.114	0.070
gcw12Δ	0.280	0.212	0.115	0.188	0.154	0.066	ku70Δ gcw39Δ	0.269	0.211	0.097	0.093	0.095	0.051
gcw13Δ	0.316	0.214	0.165	0.212	0.207	0.185	ku70Δ gcw42Δ	0.303	0.229	0.105	0.091	0.071	0.048
gcw14Δ	0.276	0.167	0.101	0.126	0.100	0.065	ku70Δ gcw43Δ	0.278	0.166	0.097	0.097	0.081	0.049
gcw15Δ	0.339	0.204	0.124	0.148	0.113	0.050	ku70Δ gcw45Δ	0.280	0.230	0.100	0.133	0.109	0.053
gcw16Δ	0.282	0.222	0.130	0.103	0.186	0.060	ku70Δ gcw46Δ	0.265	0.182	0.105	0.095	0.094	0.058
gcw17Δ	0.283	0.220	0.166	0.215	0.189	0.168	ku70Δ gcw48Δ	0.282	0.206	0.089	0.121	0.106	0.045
gcw19Δ	0.302	0.180	0.181	0.237	0.265	0.186	ku70Δ gcw49Δ	0.273	0.215	0.096	0.094	0.111	0.050
gcw21Δ	0.287	0.194	0.167	0.214	0.228	0.160	ku70Δ gcw51Δ	0.296	0.219	0.093	0.114	0.124	0.048
gcw22Δ	0.308	0.223	0.155	0.222	0.192	0.176	ku70Δ gcw52Δ	0.245	0.188	0.139	0.094	0.064	0.049
gcw61Δ	0.309	0.216	0.115	0.130	0.123	0.082	ku70Δ gcw53Δ	0.273	0.197	0.105	0.126	0.081	0.063
GS115 ku70Δ	0.258	0.197	0.093	0.118	0.090	0.050	ku70Δ gcw54Δ	0.252	0.203	0.127	0.084	0.076	0.043
ku70Δ gcw3Δ	0.226	0.212	0.088	0.075	0.065	0.066	ku70Δ gcw56Δ	0.250	0.189	0.110	0.085	0.073	0.057
ku70Δ gcw8Δ	0.255	0.221	0.061	0.090	0.103	0.046	ku70Δ gcw58Δ	0.295	0.240	0.120	0.085	0.091	0.070
ku70Δ gcw24Δ	0.270	0.202	0.091	0.121	0.112	0.050	ku70Δ gcw59Δ	0.251	0.181	0.127	0.076	0.059	0.035
ku70Δ gcw25Δ	0.265	0.212	0.092	0.087	0.118	0.047	ku70Δ gcw60Δ	0.275	0.208	0.118	0.116	0.104	0.067
ku70Δ gcw26Δ	0.264	0.185	0.101	0.119	0.098	0.047

### Remodeling of cell wall polysaccharides in the deletion library

**Fig. 2:**
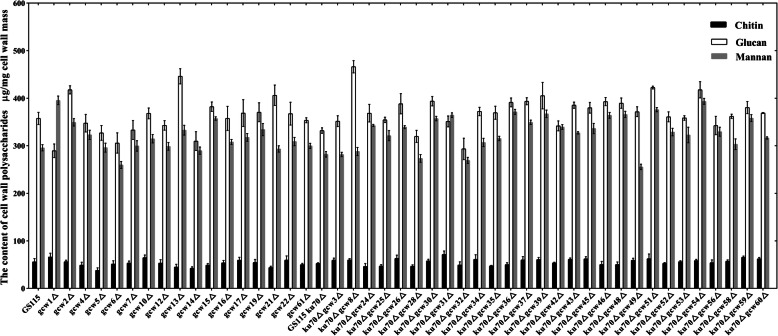
Analysis of the cell wall polysaccharide content of strain GS115 and the deletion strains after acid (H_2_SO_4_) treatment and quantification by high-performance ionic chromatography coupled to pulse amperometry detection

Cell wall polysaccharides mainly contain polymers of mannose, glucose and N-acetylglucosamine, but the synthesis of cell wall polysaccharides is also dynamic and shifts to facilitate the loosening and strengthening of the cell wall. A lack of GPI proteins may lead to the remodeling of yeast cell wall polysaccharides. The content of cell wall polysaccharides in the deletion library was measured, and changes in cell wall chitin, dextran or mannan were observed in Fig. 2.

For strain ku70Δ gcw51Δ, the contents of glucan and mannan increased by 27 and 33%, respectively. For strain ku70Δ gcw54Δ, the contents of glucan and mannan increased by 26 and 39%, respectively. In addition, the glucan content of strain ku70Δ gcw8Δ increased by 40%, and the mannan content of strain gcw1Δ increased by 34%. These results suggest that the deletion of these GPI proteins leads to remodeling of the yeast cell wall polysaccharide.

### Screening surface hydrophobic strains in deletion library strains as hosts to obtain recombinant strains with high hydrolytic activity

The surface hydrophobicity of the strains in the deletion library is listed in Table 3. Among all the deletion strains, the strains gcw13Δ, gcw22Δ, ku70Δ gcw30Δ, and ku70Δ gcw53Δ showed higher surface hydrophobic properties. Moreover, the protein GRAVY value was calculated by the sum of hydropathy values of all amino acids divided by the protein length. After identifying the strains with higher surface hydrophobic properties, some deleted GPI proteins, such as GCW13, GCW22, GCW30 and GCW53, were found to correspond to hydrophobic strains that seemed to have stronger hydrophilic properties by the GRAVY calculator.

**Table 3:** The surface hydrophobicity of deletion library strains and the GRAVY values of deleted GPI proteins

Strains	hydrophobicity	GRAVY*	Strains	hydrophobicity	GRAVY
GS115	0.24 ± 0.07	–	ku70Δ gcw28Δ	0.44 ± 0.10	0.02
gcw1Δ	0.18 ± 0.11	−0.14	ku70Δ gcw30Δ	0.49 ± 0.03	−0.73
gcw2Δ	0.38 + 0.07	−0.21	ku70Δ gcw31Δ	0.20 + 0.02	−0.11
gcw4Δ	0.43 ± 0.04	0.21	ku70Δ gcw32Δ	0.49 ± 0.10	0.23
gcw5Δ	0.34 ± 0.07	0.06	ku70Δ gcw34Δ	0.28 ± 0.07	−0.22
gcw6Δ	0.34 ± 0.02	−0.42	ku70Δ gcw35Δ	0.13 + 0.01	− 0.05
gcw7Δ	0.33 ± 0.09	−0.17	ku70Δ gcw36Δ	0.37 ± 0.03	−0.56
gcw10Δ	0.44 ± 0.00	−0.28	ku70Δ gcw37Δ	0.32 ± 0.05	0.20
gcw12Δ	0.25 + 0.04	0.16	ku70Δ gcw39Δ	0.42 ± 0.05	−0.12
gcw13Δ	0.47 ± 0.00	−0.50	ku70Δ gcw42Δ	0.27 + 0.06	0.13
gcw14Δ	0.43 + 0.09	0.20	ku70Δ gcw43Δ	0.49 ± 0.01	−0.08
gcw15Δ	0.45 ± 0.03	−0.32	ku70Δ gcw45Δ	0.36 ± 0.08	−0.64
gcw16Δ	0.17 + 0.05	−0.62	ku70Δ gcw46Δ	0.18 + 0.01	−0.22
gcw17Δ	0.14 + 0.02	−0.17	ku70Δ gcw48Δ	0.27 + 0.02	−0.26
gcw19Δ	0.20 + 0.00	0.13	ku70Δ gcw49Δ	0.31 ± 0.04	−0.34
gcw21Δ	0.27 + 0.05	0.03	ku70Δ gcw51Δ	0.30 ± 0.02	−0.20
gcw22Δ	0.57 ± 0.09	−0.68	ku70Δ gcw52Δ	0.18 ± 0.06	−0.49
gcw61Δ	0.45 ± 0.02	0.55	ku70Δ gcw53Δ	0.48 ± 0.09	−0.37
GS115 ku70Δ	0.16 ± 0.03	–	ku70Δ gcw54Δ	0.25 + 0.02	−0.21
ku70Δ gcw3Δ	0.28 ± 0.11	−0.54	ku70Δ gcw56Δ	0.32 ± 0.07	0.05
ku70Δ gcw8Δ	0.29 + 0.05	−0.04	ku70Δ gcw58Δ	0.35 + 0.03	−0.27
ku70Δ gcw24Δ	0.21 ± 0.04	−0.13	ku70Δ gcw59Δ	0.26 + 0.03	−0.36
ku70Δ gcw25Δ	0.32 ± 0.07	0.01	ku70Δ gcw60Δ	0.42 + 0.01	−0.08
ku70Δ gcw26Δ	0.22 ± 0.06	−0.18			

*GRAVY* Grand average of hydropathicity

**Fig. 3:**
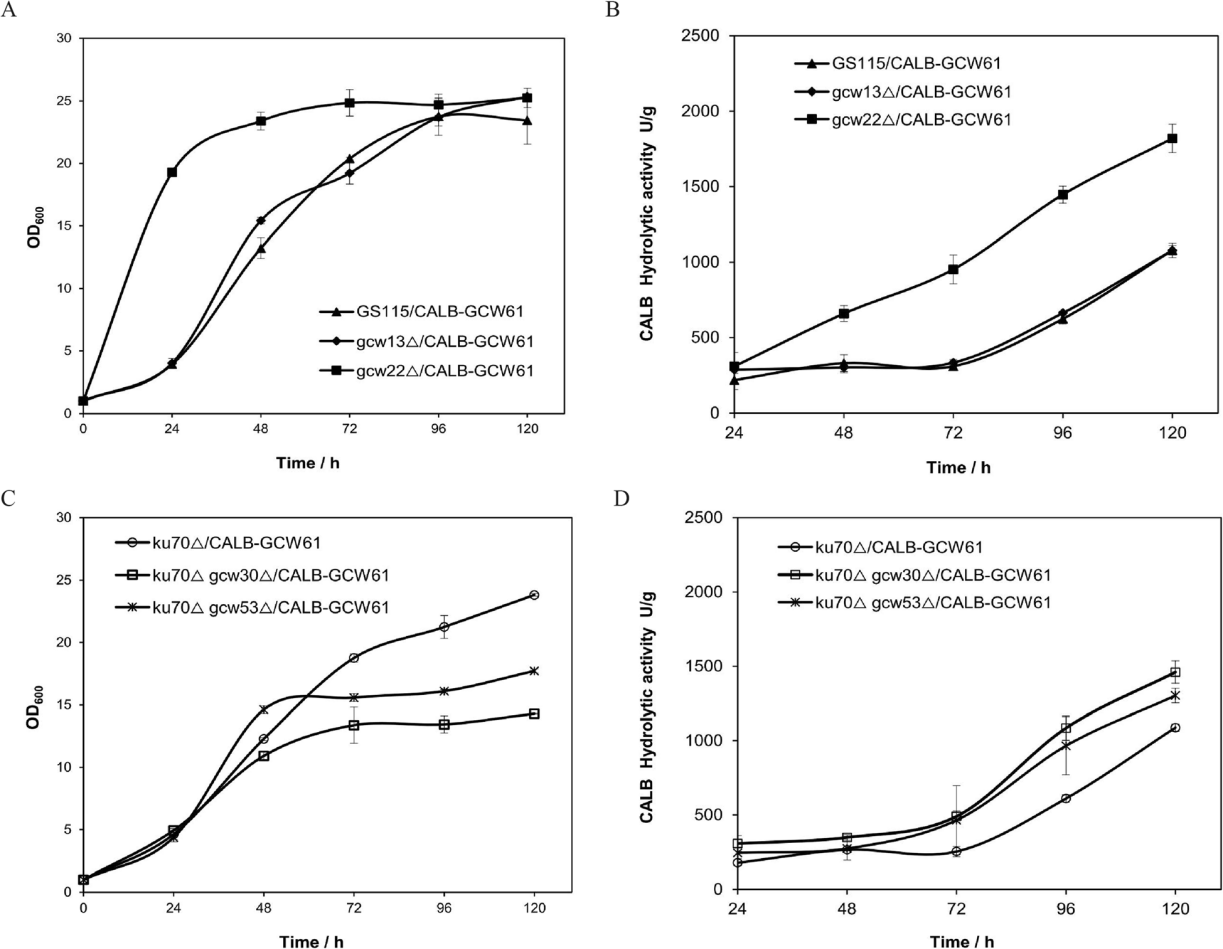
Growth characteristics and hydrolytic activity of recombinant strains. Recombinant Cells were induced to express CALB in BMMY medium containing 1% (v/v) methanol for 120 h. The hydrolytic activity was measured using pNPB as the substrate. **a** and **c**, Growth curves of deletion strains. **b** and **d**, CALB hydrolytic activity curves of deletion strains. The control strains were GS115/CALB-GCW61 and ku70Δ/CALB-GCW61

To explore the application potential of these screened hydrophobic yeast cells, the recombinant plasmid pZCALB-GCW61 was constructed. Then, the plasmid was transformed into strains GS115 and GS115 ku70Δ as control strains. The fermentation curves are shown in Fig. 3. The growth of strain gcw22Δ/CALB-GCW61 was faster than that of other strains in the first 72 h and then soon reached a plateau. The specific growth rate, μ, of strain gcw22Δ/CALB-GCW61 was 0.33 h^− 1^, corresponding to an increase of 23% compared with strain GS115/CALB-GCW61 (0.27 h^− 1^). Strain gcw13Δ/CALB-GCW61 maintained a similar growth rate as the control strain. The growth rate of the other three strains, ku70Δ gcw30Δ/CALB-GCW61 and ku70Δ gcw53Δ/CALB-GCW61, slowed down significantly after 48 h. The lipase hydrolysis activity of all the recombinant strains increased except strain gcw13Δ/CALB-GCW61. In particular, the hydrolysis activity of strain gcw22Δ/CALB-GCW61 increased by 69% after fermentation for 120 h. The surface hydrophobicity of the recombinant strain was also detected. This result was consistent with the enzyme activity results, and strain gcw22Δ/CALB-GCW61 showed the highest surface hydrophobicity. Although strain ku70Δ gcw30Δ/CALB-GCW61 had the same surface hydrophobicity, the lipase activity did not significantly change (Table 3).

## Discussion

Among the 50 putative GPI proteins in *P. pastoris*, more than 70% of them have unknown physiologic functions. Using the Cre/loxP system, a library of 45 deletion strains representing almost 90% of the candidate GPI genes was constructed. This is the largest library of GPI protein deletions so far, and this library is a significant step towards unveiling the roles of all the GPI proteins of *P. pastoris*. In this study, the deletion library was subjected to a series of phenotypic tests, and some strains with potential applications were found and evaluated.

The methylotrophic yeast *P. pastoris* is currently a powerful system for the production of recombinant heterogeneous proteins [18]. The control of the methanol concentration in an appropriate range can promote yeast growth and protein expression. However, higher methanol concentrations often lead to the accumulation of formaldehyde, retard growth and decrease the observed biomass yield [19, 20]. However, a lower methanol concentration means less carbon source, resulting in a decrease in protein production. Therefore, improving the tolerance of the strain to methanol and increasing the rate of methanol utilization for *P. pastoris* have been in great demand. The strategy can also shorten the period of yeast culture, increase the production intensity and reduce the production cost. Five strains in this deletion library, gcw13Δ, gcw17Δ, gcw19Δ, gcw21Δ, and gcw22Δ, showed obvious methanol tolerance characteristics in YPM and BMMY media supplemented with different methanol concentrations. It could be speculated that these deletion strains triggered remodeling of cell wall protein composition and led to an increase in cell tolerance to methanol because the cell membrane and cell wall were major targets of methanol toxicity [21]. Studies have demonstrated that GCW13 suppresses the expression of GAP1, which encodes a general amino acid permease, and facilitates the endocytosis of GAP1 in methanol. The deletion of the GCW13 gene derepresses GAP1-dependent uptake of amino acids and shows better methanol tolerance [22]. In *C. glutamicum*, increased methanol tolerance may be relevant to two point mutations: one leading to the amino acid exchange A165T of the O-acetylhomoserine sulfhydrolase MetY and the other leading to shortening of the CoA transferase Cat (Q342*) [23]. In *S. cerevisiae*, a large number of genes required for methanol tolerance are attributed to vacuolar function, transcription and some unknown function [24]. In addition to the protein GCW13, the physiologic functions of other GPI proteins, GCW17, GCW19, GCW21 and GCW22, need to be explored further.

Mannoproteins and β-glucans represent the majority of the polysaccharides in the yeast cell wall. In recent years, yeast polysaccharides have been proven to be immunostimulant molecules for human and animal health due to their ability to enhance and stimulate the immune system, presenting antitumor, antiinflammatory, antimicrobial, wound healing, weight loss, and antidiabetic properties [25, 26]. β-1,3-Glucan is also a valuable microbiological binder of mycotoxins that decreases their toxic effects and mediates their removal from media [27, 28]. Mannoproteins were reported to contribute to wine quality, including provide protection against protein and tartaric instability, reduction of astringency, retention of aromatic compounds, and increased body and mouth feel, which are especially appreciated in red wines [29]. Therefore, interest in industrial production of cell wall polysaccharides has been increasing. At present, the usual methods used to increase the content of yeast polysaccharides include the optimization of fermentation conditions, regulation of cell osmotic pressure or mutagenesis breeding [30–32].

In this deletion library, the remodeling of cell wall polysaccharides resulting from certain GPI protein knockouts led to some promising strains, such as the strain ku70Δ gcw8Δ with a higher glucan content and strain gcw1Δ with a higher mannan content. It is also surprising that the glucan and mannan contents of two strains, ku70Δ gcw51Δ and ku70Δ gcw54Δ, were increased. The gene GCW8 was predicted to be a mannosidase that was capable of catalyzing the hydrolysis of mannose nonreducing residues. In this study, the gene GCW8 was redundant because there were no obvious abnormalities in cell morphology or growth with the strain gcw8Δ, but the gene was involved in the regulation of cell wall structure synthesis. The gene GCW8 has higher homology with the DCW1 and DFG5 proteins of *S. cerevisiae*, both of these proteins play important roles in cell wall protein cross-linking to the cell wall reticular structure [33]. In *S. cerevisiae*, there were no abnormal changes in cell growth or cell morphology when these two genes were knocked out individually [34, 35]. The strain dcw1Δ was hypersensitive to zymolyase, which is a cell wall-digesting enzyme, leading to defects in cell wall structure. This result indicated that the gene dcw1 is involved in the regulation of cell wall structure. Further studies suggested that both Dcw1p and Dfg5p are GPI-anchored membrane proteins that are required for normal biosynthesis of the cell wall [36]. Abhiram knocked out the genes DCW1 and DFG5 in *Neurospora crassa* separately, the cell wall proteins would be slight released into the medium. When the two genes were knocked out simultaneously, the release of cell wall proteins into the medium greatly increased, the content of mannan in the cell wall decreased from 14.8 to 2.3%, and the content of glucan increased from 72.8 to 92.6% [33]. The gene GCW1, also known as GAS1 in *P. pastoris*, is a β-1,3-glucanosyltransferase that catalyzes a transglycosylation with β-1,3-glucan as a substrate. In *S. cerevisiae*, the loss of Gas1p resulted in the release of β-1,3-glucan into the medium and an increase in chitin and mannan content in the cell wall. This result was consistent with our finding that the content of mannan was increased by 34% in strain gcw1Δ. It is speculated that the higher mannan content is due to a higher expression of cell wall mannoproteins, which might be part of a general response of the cell to cell wall defects in an attempt to prevent cell lysis [37]. In *P. pastoris*, the morphology of gas1Δ was identical to that of *S. cerevisiae* [38]. The genes GCW51 and GCW54 have been little studied, and their physiologic functions were unknown, but according to the above data, both genes must have at least one function, e.g., playing a structural role, contributing to the biosynthesis or assembly of the major cell wall components, or playing a part in cell wall remodeling. Their detailed features will be further explored in the future.

The display of lipase on the cell surface as a whole-cell catalyst is currently a very active topic in organic bioconversion. The hydrophobic environment is commonly believed to be well suited for lipase catalysis because of the “interfacial activation” mechanism [39, 40], but the surfaces of yeast cells are commonly relatively hydrophilic due to the presence of various polysaccharides and proteins. This possibly causes contact resistance between enzyme molecules and hydrophobic substrates [41], and hydrophilic byproducts such as glycerol and water are absorbed on the cell surface and form a hydrophilic layer, leading to cell aggregation and poor dispersibility [42]. To alleviate these problems, hydrophobins are displayed on the *P. pastoris* cell surface; hydrophobins modify the relative hydrophilic properties and improve the catalytic activities of lipase to some extent [43]. Other methods, including coating with ionic liquids and adding decane as a hydrophobic carbon source during fermentation, show higher surface hydrophobicity. Herein, the strains gcw13Δ, gcw22Δ, ku70Δ gcw30Δ, and ku70Δ gcw53Δ attracted attention for their higher surface hydrophobicity. Bioinformatics analysis revealed that the amino acid sequences of the deleted proteins GCW13, GCW22, GCW30, and GCW53 were all highly hydrophilic. Hsu et al. observed clinical isolates of *S. cerevisiae* with relatively hydrophobic cell surfaces and found that the change in cell wall protein composition was related to the hydrophobicity. The abundances of Scw10p, Pst1p, and Hsp150p/Pir2p, which are all cell wall proteins in the clinical isolates, were at least two-fold higher than those in the S288c lab strain [44]. It is speculated that the absence of these proteins leads to the remodeling of cell wall structures, including the cell wall protein quantity and type, and finally the distribution of cell surface hydrophobicity.

To investigate the application potential of the strains with higher surface hydrophobicity, the lipase CALB was displayed with the same anchor, GCW61, on these above strains. Interestingly, the hydrolytic activities of strains gcw22Δ/CALB-GCW61, ku70Δ gcw30Δ/CALB-GCW61 and ku70Δ gcw53Δ/CALB-GCW61 increased by 69, 34 and 20%, respectively, but there was no obvious change in the lipase hydrolytic activity in strain gcw13Δ/CALB-GCW61. We inferred that lipases immobilized on the cell surfaces of these hydrophobic yeast were similar to those immobilized on other hydrophobic carriers; the more hydrophobic environment kept the conformations of the displayed CALB open, and finally, the activities of CALB were affected. It is also desirable to exploit strains with high surface hydrophobicity in other fields, such as wastewater treatment [45] and bioremediation of contaminated sites [46].

## Conclusions

A total of 45 *P. pastoris* strains with deletions in genes encoding predicted GPI proteins were constructed and showed different characteristics. Among them, some deletions appeared to increase tolerance to methanol, some deletions showed an increase in wall polysaccharides, and others showed surface hydrophobicity. These promising deletion strains not only showed potential applications as production strains but also offered more possibilities to study more functions of GPI proteins, especially putative ones.

## Methods

### Strains, plasmids and culture conditions

*P. pastoris* GS115, which was used as a parental strain for gene disruption, and the plasmid pPICZαA, which was used as the vector, were both purchased from Invitrogen (Carlsbad, CA, USA). The yeast cells were grown in media with different carbon sources for growth analysis, including complex medium (1% (w/v) yeast extract, 2% (w/v) peptone) supplemented with 2% (w/v) glucose (YPD), 2% (w/v) glycerol (YPG), and 1, 2%, or 3% (w/v) methanol (YPM). The buffered glycerol complex (BMGY) contained 1% (w/v) yeast extract, 2% (w/v) peptone, 1.34% (w/v) yeast nitrogen base, 1% (v/v) glycerol and 50 mM potassium phosphate buffer. The buffered methanol complex (BMMY) was the same as BMGY but 1% glycerol was substituted with methanol. The BMGY and BMMY culture media were used for growth and induction studies, respectively. All yeast strains were cultured at 30 °C.

### Construction of the GPI protein deletion library

Plasmid pPICZαA was used as a vector to construct plasmid pPICZC according to the method described by Pan et al. [47]; pPICZC carries the expression cassette of Cre recombinase and the selective marker gene for zeocin resistance. Using the plasmid pPICZC as template and P3 with the lox71 site and P4 with the lox66 site as primers, the modular *lox71*-Cre-ZeoR-*lox66* (CORE) cassette was amplified by PCR. To delete a target gene, the upstream (by primers P5/P6) and downstream (by primers P7/P8) homologous arms of the target gene were individually amplified from *P. pastoris* GS115 genomic DNA by PCR. The reverse primer amplifying the Up-arm fragment and the forward primer amplifying the Down-arm fragment shared 30–40 nucleotides with the CORE cassette. The gene disruption cassettes (Up homologous-lox71-Cre-ZeoR-lox66-Down homologous) were constructed by fusing the CORE cassette with two homologous fragments flanking the region. The disruption cassettes were introduced into the parental strain GS115. After the clones were screened on YPDSZ (YPD plus 1 M sorbitol and 50 mg/L zeocin) plates and verified by genomic PCR analysis, the Cre-mediated recombination between *lox*71 and *lox*66 was subsequently removed through a methanol induction step in YPM medium using two outer primers, P9/P10. To improve the homologous recombination efficiency, the endogenous gene KU70 homologue in GS115 as a host was deleted using this Cre/loxP system. Thereafter, the potential GPI-anchored proteins selected from the ORFs of the *P. pastoris* GS115 genome were knocked out according to the method of Zhang et al. [11]; thus, a single GPI gene deletion library was obtained. The sequences of all the primers are provided in Supplementary Table S1.

### Growth analysis on different carbon sources

The deletion strains in GPI-anchored proteins were cultured in 96-well master plates at 30 °C to obtain a growth curve. The cultures included YPG medium with glycerol as the carbon source, YPD medium with glucose as the carbon source and YPM medium with methanol as the carbon source. To characterize cell growth, the specific growth rate, μ, was estimated using graphical methods from a linear regression of the natural logarithm of the number of viable cells versus time. It was calculated from the following equation: μ = (In N_2_ - InN_1_)/(t_2_-t_1_). The growth of strains in BMMY medium with methanol added every 24 h was recorded.

### Measurement of polysaccharide content in the cell wall

The cell wall polysaccharide was measured as described by François J M with some modifications [48]. To extract cell wall polysaccharides, cells were collected and resuspended in 1 mL of cold water. After centrifugation at 10,000×g for 5 min, the cell pellet was resuspended in 750 μL of buffer TE, and 0.73 g of acid-washed glass beads were added. Disruption of the cells was performed with a mechanical bead beater set at full speed for eight 30 s periods alternating with 30 s intervals on ice. The glass beads were washed at least five times with 1 mL of TE buffer by vortexing the cell suspension briefly and then spinning down the beads (500 g for 1 min) after each wash. When the washing solution became limpid, most of the cell debris was no longer bound to the beads. All the washing solutions were centrifuged at 4800×g for 15 min, and the cell wall fragments were then freeze-dried. Finally, 10 mg cell wall fragments were weighed accurately, wetted with 75 μL of 72% H_2_SO_4_ solution for 3 h at room temperature and diluted to 2 N H_2_SO_4_ for 4 h at 100 °C. The acid solution was neutralized with saturated Ba (OH)_2_ until the pH was neutral, and the final volume was adjusted to 20 mL with Milli-Q water. The solution was centrifuged at 4800×g for 15 min at 4 °C. One milliliter of the supernatant was taken for HPIC measurement according to François J M [48].

### Measurement of cell surface hydrophobicity

The hydrophobic nature of the outermost surface of the yeast cells was determined by microbial adhesion to hydrocarbon (MATH), as described by Hama et al. [41] and modified by Wang et al. [43]. In this method, the proportion of yeast cells passing into the n-butyl alcohol reflects the adsorption of the yeast cells to the organism. The cells were washed twice and resuspended in PBS to an OD_600_ of 2, and the accurate value was defined as A_1_. A 2 mL aliquot of butyl alcohol was then added to 2 ml of the cell suspension, vortexed for 30 s and allowed to stand for 3 min to enable the complete separation of the two phases. The lower aqueous phase was measured at 600 nm and defined as A_2_. A_1_ and A_2_ were measured using a spectrophotometer (BIO-RAD, USA). Hydrophobicity was then given as a percentage calculated from the following equation: hydrophobicity (%) = (A_1_ − A_2_)/A_1_. The average hydrophobicity expressed as the grand average of hydropathy (GRAVY) value [49] for identified proteins and peptides was calculated using Prot-Param software available at https://web.expasy.org/protparam/.

### Analysis of CALB hydrolytic activity

The plasmid pZCALB-GCW61 containing mature CALB cDNA [50] was transformed into strains GS115 and GS115 ku70Δ as the control strains. CALB hydrolytic activity was assayed using a modified method [11]. The substrate *P*-nitrophenyl butyrate (pNPB; Sigma, St. Louis, MO, USA), which was the substrate, was emulsified by sonication in ultrapure water containing 0.5% Triton X-100, resulting in a final concentration of 25 mM. The 1 mL reaction system contained 940 μL of Tris-HCl buffer (50 mM, pH 8.0), 50 μL of pNPB, and 10 μL of cell supernatant with the appropriate dilution to ensure the absorbance was in a reasonable range. Then, the system was incubated for 5 min at 45 °C. Finally, the assay mixture was centrifuged at 6000×g for 1 min. Using a kinetic microplate reader (Molecular Devices, Sunnyvale, CA, USA), the absorbance of 200 μL supernatant was measured at 405 nm with a 96-well plate. The hydrolytic activity of CALB was defined as the amount of enzyme required to release 1 μmol pNP per min under the assay conditions. Average values were generated from triplicates of each sample.

## Supplementary information

**Additional file 1: Table S1.** Primers used in this study.

## Data Availability

The datasets used and analysed during the current study are available from the corresponding author on reasonable request.

## Abbreviations

GPI: Glycosylphosphatidylinositol
CALB: *Candida antarctica* lipase B
CWP: Cell wall protein
CWI: Cell wall integrity
NHEJ: Non-homologous-end-joining
MATH: Microbial adhesion to hydrocarbon
pNPB: p-nitrophenyl butyrate
pNP: p-nitrophenol
