# Supplementary material for: Construction and screening of a glycosylphosphatidylinositol protein deletion library in Pichia pastoris
Source: BMC Microbiol. 2020 Aug 24;20:262. doi: 10.1186/s12866-020-01928-y (PMC7446130; doi:10.1186/s12866-020-01928-y)
Supplement: Supplementary file 1 — Additional file 1: Table S1. Primers used in this study. [file 12866_2020_1928_MOESM1_ESM.docx]

Table S1. Primers used in this study.

| Primers | Sequences (5’– 3’) |
| --- | --- |
| P3 | TACCGTTCGTATAGCATACATTATACGAAGTTATAGATCTAACATCCAAAGACG |
| P4 | TACCGTTCGTATAATGTATGCTATACGAAGTTATGTCTCCAGCTTGCAAATTAA |
| P5-GCW1 | CATGCATACGAAAGGAATGAAAAAC |
| P6-GCW1 | AGATCTATAACTTCGTATAATGTATGCTATACGAACGGTA ATTTTGATTATCTTTGTGAGACGCG |
| P7-GCW1 | GGAGACATAACTTCGTATAGCATACATTATACGAACGGTAGCGGTTCACATTAATTAAAGGCACA |
| P8-GCW1 | TCTATTGGTTGAAAAAGACCGAGGG |
| PC9-GCW1 | CATAATGTTTGGCTTAGCATCCTGG |
| P10-GCW1 | GGACAACATTGCTTTGGGGTGATAC |
| P5-GCW2 | CCTCCGAAGTCTAAGCCTCCATTTT |
| P6-GCW2 | AGATCTATAACTTCGTATAATGTATGCTATACGAACGGTA TGCTTTGGGGTGATACAAAGAGTGG |
| P7-GCW2 | GGAGACATAACTTCGTATAGCATACATTATACGAACGGTAGACCTCAGGATTCACTTACACTCTT |
| P8-GCW2 | GCGAATAAGCGAGAGTATAGAGGAT |
| PC9-GCW2 | AACAGTTACGCTCAAAGAGAGGGTG |
| P10-GCW2 | AGGATGATAATACCGGATTGGGAGC |
| P5-GCW3 | CTGGTGGAACAAGACGGTGTAAAAG |
| P6-GCW3 | AGATCTATAACTTCGTATAATGTATGCTATACGAACGGTA ATGCCATCAAGGAGAGAGTTAGGGT |
| P7-GCW3 | GGAGACATAACTTCGTATAGCATACATTATACGAACGGTACTCTTGCAGCCATTATTTCCTACCT |
| P8-GCW3 | GATCAAACTACGAAGATCGCCATCT |
| PC9-GCW3 | TTCTACATCGTCTCCCAAACTATCC |
| P10-GCW3 | AGAATCTCATAGTTTGGACTTGGCT |
| P5-GCW4 | CGAACCAGGTGTGAAAGAATAAA |
| P6-GCW4 | AGATCTATAACTTCGTATAATGTATGCTATACGAACGGTA GAAGCAACGACTGGAATCAAGAA |
| P7-GCW4 | GGAGACATAACTTCGTATAGCATACATTATACGAACGGTAGGTGCTGTTATGGGTTACCTCTT |
| P8-GCW4 | CCGAAAGTGATGCCTCCAATAAC |
| PC9-GCW4 | GAGGCAAGCACCAAGACTTTCAT |
| P10-GCW4 | TGGTTCGCTTGATTCGTGTTTAG |
| P5-GCW5 | GCAAAAGAACCAGGCATTGACTA |
| P6-GCW5 | AGATCTATAACTTCGTATAATGTATGCTATACGAACGGTA GGCGGAACCTAAAAAGAGAGATC |
| P7-GCW5 | GGAGACATAACTTCGTATAGCATACATTATACGAACGGTAGCTGCTTTGGTCTTGTAAGTTTG |
| Primers | Sequences (5’– 3’) |
| P8-GCW5 | ATGATGTGGAGACTTTAACCGCT |
| PC9-GCW5 | AGTTTGTTAGCCAATCAGTTCCC |
| P10-GCW5 | TTTATCCCTTGTTTTATGCGAGC |
| P5-GCW6 | CGAAAGCCTACATCCATAGAGTG |
| P6-GCW6 | AGATCTATAACTTCGTATAATGTATGCTATACGAACGGTA TTCAGGCGAAAATAATAGCAACG |
| P7-GCW6 | GGAGACATAACTTCGTATAGCATACATTATACGAACGGTACTTTCAGCTGGTTTCCTTACTACTA |
| P8-GCW6 | GAAGTATGGTTGAGTAGGATGGAAG |
| PC9-GCW6 | AATATTTACCAGGACTTGTGCCCAT |
| P10-GCW6 | TGGATGAAATTTGGCAGACATAACA |
| P5-GCW7 | AACCGTTATTCAGTGTCTGCAAA |
| P6-GCW7 | AGATCTATAACTTCGTATAATGTATGCTATACGAACGGTA AGCTGGATCACAAACATTCGGTA |
| P7-GCW7 | GGAGACATAACTTCGTATAGCATACATTATACGAACGGTACCCTGCATTATTGCCTTCCTACT |
| P8-GCW7 | AATCGTGATCAAAAGGGCGTAGT |
| PC9-GCW7 | TAGCATTAGGCTGTGAGTCTGGC |
| P10-GCW7 | CGACCTTGCCGTTGTTCTTACTT |
| P5-GCW8 | CCACAACACCTTTACTACGCTTC |
| P6-GCW8 | AGATCTATAACTTCGTATAATGTATGCTATACGAACGGTA ATTTTGTTGAATAGAAGGAAGGG |
| P7-GCW8 | GGAGACATAACTTCGTATAGCATACATTATACGAACGGTACGGTGCCATTTGGTTACTCTTAT |
| P8-GCW8 | ATACGCAGAGAGTCGTGCTTCAA |
| PC9-GCW8 | TCCCTGACCAGCAATCACATAAG |
| P10-GCW8 | AAGAGCCGCACAGAGAATGAAAT |
| P5-GCW10 | GAACCAATTGTCCAATCTGTCTTAG |
| P6-GCW10 | AGATCTATAACTTCGTATAATGTATGCTATACGAACGGTA CAGCAATAGAGGGGATATCAAGTTC |
| P7-GCW10 | GGAGACATAACTTCGTATAGCATACATTATACGAACGGTACTAGGACTACCGTTGATTCTGGC |
| P8-GCW10 | CACTCTCAGCTAACAGGGCCAAT |
| PC9-GCW10 | AGGACAAGGATCTGGATGAGTTC |
| P10-GCW10 | GACCTGTTGTCGGTAACCATTTG |
| P5-GCW12 | GAACGAATAAAACTCGCTCTCAA |
| P6-GCW12 | AGATCTATAACTTCGTATAATGTATGCTATACGAACGGTA CAACAACAGAGGTAGAAGCGAAA |
| P7-GCW12 | GGAGACATAACTTCGTATAGCATACATTATACGAACGGTAGCTTTGGCTGTTGGTCTGATCTA |
| Primers | Sequences (5’– 3’) |
| P8-GCW12 | GGTGAACAGCGTAGGCAAAGTCT |
| PC9-GCW12 | AACCCCTATTGGAGTTGAGAGAA |
| P10-GCW12 | TCTTTGATGAGGCGGTCCAGTAA |
| P5-GCW13 | TTCTGTCATAGTGTCTACCCCTCAA |
| P6-GCW13 | AGATCTATAACTTCGTATAATGTATGCTATACGAACGGTA GCAATACCTACGCTTCTCATAATCT |
| P7-GCW13 | GGAGACATAACTTCGTATAGCATACATTATACGAACGGTAGGTCATGTACAGTGTTTTGTTCGTG |
| P8-GCW13 | TATGGAGTTTGTATTGTTCCCCGTT |
| PC9-GCW13 | TGGAGAACCAAGAATCACTGAAACA |
| P10-GCW13 | CCTATCTAATCACCCATTGTCACCT |
| P5-GCW14 | GCCACGTTTTAACCTTAAAGTCGTTC |
| P6-GCW14 | AATGTATGCTATACGAACGGTATTTTGTTGTTGAGTGAAGCGAGTG |
| P7-GCW14 | AGCATACATTATACGAACGGTAGTATCTCCAGTCGTTTAGATTG |
| P8-GCW14 | TTTAATACGTAGGCACTCCAGTTCCACAG |
| PC9-GCW14 | AGTTCACAATCAAGGTGAGTCCAG |
| P10-GCW14 | GAAACCGTCCCCGATCACCGAGATA |
| P5-GCW15 | GGCTTTGAGAAGGTAATCTTGAACA |
| P6-GCW15 | AGATCTATAACTTCGTATAATGTATGCTATACGAACGGTA ACTCAGTAACACCAAGCCAACAAAC |
| P7-GCW15 | GGAGACATAACTTCGTATAGCATACATTATACGAACGGTAAAGCCCTTCCCTTCTCTCTTTCTAC |
| P8-GCW15 | CCTATTTGATTGGACCTATGTTGGA |
| PC9-GCW15 | CTAATGGTTCAGGTTATGGAAATGC |
| P10-GCW15 | GGCTGGAGTAATATTTGATGGATCT |
| P5-GCW16 | GTATAATATTAGGTGCCTTTGTCGC |
| P6-GCW16 | AGATCTATAACTTCGTATAATGTATGCTATACGAACGGTA TAGTTGTTTGTGTGGTTTGGGTAGT |
| P7-GCW16 | GGAGACATAACTTCGTATAGCATACATTATACGAACGGTATGATCACTTATGAACACTTCTCCTG |
| P8-GCW16 | CAAAAGCTCGGCTCTATAAGTACTA |
| PC9-GCW16 | GAGACGGTGCATGTATTAACTTTGA |
| P10-GCW16 | GTATAATATTAGGTGCCTTTGTCGC |
| P5-GCW17 | GTTTAGATTACTTTTAGTTTTCGCGG |
| P6-GCW17 | AGATCTATAACTTCGTATAATGTATGCTATACGAACGGTA CTGTGAATTTTCATTTTCAGTTCTGG |
| P7-GCW17 | GGAGACATAACTTCGTATAGCATACATTATACGAACGGTACTTAGGAGCAGCCGCAGTTTTAC |
| Primers | Sequences (5’– 3’) |
| P8-GCW17 | CAATAACAAGCAGCAAATCGCCT |
| PC9-GCW17 | GCACCAGATACATTCTACAGGGA |
| P10-GCW17 | CCAGCATCCTGAGTAAGCATTAT |
| P5-GCW19 | GATTTGTAAACATCATCGCCAGG |
| P6-GCW19 | AGATCTATAACTTCGTATAATGTATGCTATACGAACGGTA CTTAATGCGAGAGTTGCACCGAT |
| P7-GCW19 | GCTGGTCTCTTCGTCGCTATGTT |
| P8-GCW19 | GCATGATGGTCAATTCGGCACTA |
| PC9-GCW19 | CCCTGGTCCGTTACTCTCCTCTA |
| P10-GCW19 | CAACTACTGGAGCAATTGGAGAA |
| P5-GCW21 | ATTTTGACTTCATTGGTGCTTTC |
| P6-GCW21 | AGATCTATAACTTCGTATAATGTATGCTATACGAACGGTA TTCAAGTAAGACATGGCGTTCAA |
| P7-GCW21 | GGAGACATAACTTCGTATAGCATACATTATACGAACGGTA ACTATGGTTTGTTGGTTGCAGCT |
| P8-GCW21 | TTACCAATCTTTGTTACACCCCG |
| PC9-GCW21 | CCGTCGTTTACAAGAGTGTGATC |
| P10-GCW21 | GGAGGATACTGTAGATCGGGATG |
| P5-GCW22 | GCCAAGCTGTAATAAGCCCGTAG |
| P6-GCW22 | AGATCTATAACTTCGTATAATGTATGCTATACGAACGGTA AGAAGAGGTGAATATCGATCGCA |
| P7-GCW22 | GGAGACATAACTTCGTATAGCATACATTATACGAACGGTA CTATGATATCCGGAGCAGTTGCA |
| P8-GCW22 | GCTGTCAGTGCTACGTGCTTCTT |
| PC9-GCW22 | AATCCCGTGTACGCATTCTTTCA |
| P10-GCW22 | TAATCCTGGCTTCAAAGGCTGTT |
| P5-GCW24 | GAGCATAAAGGAGAAAAAAACGC |
| P6-GCW24 | AGATCTATAACTTCGTATAATGTATGCTATACGAACGGTA ACTTCATGATTTCTTTTGGACCGc |
| P7-GCW24 | GGAGACATAACTTCGTATAGCATACATTATACGAACGGTAATGTGTTTGGCAATGGCTTTGAT |
| P8-GCW24 | ATTTCTCCATCAACCTCCAGCAC |
| PC9-GCW24 | CTCAGGTGTGACAGATATTCGGC |
| P10-GCW24 | AGATGGGTTACGACCTCTGGAAG |
| P5-GCW25 | ATGGCGAGTATGTAAACTGGCAC |
| P6-GCW25 | AGATCTATAACTTCGTATAATGTATGCTATACGAACGGTA AAGCGCCTAGCACTAAGAACAAT |
| P7-GCW25 | GGAGACATAACTTCGTATAGCATACATTATACGAACGGTA GTGTTCTTCGTTTAGATGCCCCT |
| Primers | Sequences (5’– 3’) |
| P8-GCW25 | GCAAACTGGCAGGAAGGACTAAT |
| PC9-GCW25 | TTGCGTTAGGAACATGCGCTATAGT |
| P10-GCW25 | AGTTACTCAACAAAGGGTAGGGTCG |
| P5-GCW26 | ATGGTTTCTAACTTAATCGATGCGT |
| P6-GCW26 | AGATCTATAACTTCGTATAATGTATGCTATACGAACGGTA TAGAAACAAAAGTGGCACAGGAAAT |
| P7-GCW26 | GGAGACATAACTTCGTATAGCATACATTATACGAACGGTAGTGCCCTGTTCATGTAAACTTCC |
| P8-GCW26 | CCGATATGATAGCCGCTACAGAT |
| PC9-GCW26 | GGAAAGAAAAGCAACAAGACGAG |
| P10-GCW26 | CAACTTTGAGTATTGTGGATGCC |
| P5-GCW28 | TAGTGTCCAAAAGGGGATCGCTC |
| P6-GCW28 | AGATCTATAACTTCGTATAATGTATGCTATACGAACGGTAGCGGCAGCCTGTAGGATAATAAG |
| P7-GCW28 | GGAGACATAACTTCGTATAGCATACATTATACGAACGGTAGCTGTTGGTCTGTTAGCAATCGT |
| P8-GCW28 | CAGCATGCCTGCAGTTTAACAAT |
| PC9-GCW28 | TATTGTTGCGGTGGCGATTTGAC |
| P10-GCW28 | GAGCATTCCAAAACATTGCGGTC |
| P5-GCW30 | TTCGAATGTGAGGATGAAGATGC |
| P6-GCW30 | AGATCTATAACTTCGTATAATGTATGCTATACGAACGGTATTTCTAACTGCTTGCTGAACTGG |
| P7-GCW30 | GGAGACATAACTTCGTATAGCATACATTATACGAACGGTAGCATCAGTGGTCTTGAGCGGTCT |
| P8-GCW30 | TACCGCTCTTTTCTGTGCCATCG |
| PC9-GCW30 | GTTTACAAGCCTACCATTCGTCG |
| P10-GCW30 | CTTACTTTTGCTGACCGTGCTGT |
| P5-GCW31 | ACTACACAGTTGAGCTTGTCGCG |
| P6-GCW31 | AGATCTATAACTTCGTATAATGTATGCTATACGAACGGTAGCAATGCTGAGGGCTGTCAATAC |
| P7-GCW31 | GGAGACATAACTTCGTATAGCATACATTATACGAACGGTAGTCTTTTTGCCATCTTGCTGCTC |
| P8-GCW31 | ATTCTCAGACGAGGAACTGGGAT |
| PC9-GCW31 | AAACCCGAAGAGTTGATGAGACC |
| P10-GCW31 | CCTCATCACCGTAACGCTCAAAT |
| P5-GCW32 | CAACGCCTCAAAATTCACCAATG |
| P6-GCW32 | AGATCTATAACTTCGTATAATGTATGCTATACGAACGGTAGCGATAGCCAAAAGAGATGCGAT |
| P7-GCW32 | GGAGACATAACTTCGTATAGCATACATTATACGAACGGTATCTTTAGGGTTGGCTGCTATTGC |
| Primers | Sequences (5’– 3’) |
| P8-GCW32 | GCCATCAACAAGAAGAAGCCAAT |
| PC9-GCW32 | CCTACCAGAAACTTTTCACGCAGCT |
| P10-GCW32 | TCAGCCTTGAGTCAGGAAAGATGTG |
| P5-GCW34 | ATGCACACTTTCGGTCCGATGAG |
| P6-GCW34 | AGATCTATAACTTCGTATAATGTATGCTATACGAACGGTAGAAGTTTGGATAAGCGGATGGGC |
| P7-GCW34 | GGAGACATAACTTCGTATAGCATACATTATACGAACGGTACATTTACATTTTGGGGTTTCGTCCT |
| P8-GCW34 | AGAAGAAACCGCAGGACCAACTAAG |
| PC9-GCW34 | TGACAACTTCAATAAGGGCTGGG |
| P10-GCW34 | GCTGTTCTAACCGTCCTCAATCC |
| P5-GCW35 | GGCACAGATTTACATGAAGTCCC |
| P6-GCW35 | AGATCTATAACTTCGTATAATGTATGCTATACGAACGGTACAGAGACAAAACCAACAACGCCT |
| P7-GCW35 | GGAGACATAACTTCGTATAGCATACATTATACGAACGGTATTACATCATTGGCCAGTCTGTTG |
| P8-GCW35 | CGGTGTGGTTGAATTTCTAGCTT |
| PC9-GCW35 | ACGGTGTCAACGAAAATCCAATG |
| P10-GCW35 | TCGAGGACCCAATTTATTGACAG |
| P5-GCW36 | GGTCTTGAACATTGTACAGTCGAT |
| P6-GCW36 | AGATCTATAACTTCGTATAATGTATGCTATACGAACGGTA GACAGCAGCTAGTGTTGAAAGGGC |
| P7-GCW36 | GGAGACATAACTTCGTATAGCATACATTATACGAACGGTAAGACACACTCTGGTGCTGCTAACA |
| P8-GCW36 | GTAGGCGATTGGAAAAAGAGATAG |
| PC9-GCW36 | AAGGGGTTGAAGATCACAGAGAAG |
| P10-GCW36 | TGTAGCTGGTGACCCAAATTTAGA |
| P5-GCW37 | TTGTAGCGAGTGTCATAAGCGG |
| P6-GCW37 | AGATCTATAACTTCGTATAATGTATGCTATACGAACGGTATTAGGAGCAGCATTAGCCAGTG |
| P7-GCW37 | GGAGACATAACTTCGTATAGCATACATTATACGAACGGTAAGGTATCGTTGGTGCTATTGGT |
| P8-GCW37 | CTGCCAAATCTCCTTTCGCTGT |
| PC9-GCW37 | AAAGGTGGCAAACAATGCTACT |
| P10-GCW37 | AAATCTGCCTTTGGAGGAACTT |
| P5-GCW39 | AACCCCAGAGTATTGCTTAGGC |
| P6-GCW39 | AGATCTATAACTTCGTATAATGTATGCTATACGAACGGTACAACACTAGGGCAACAGCAACT |
| P7-GCW39 | GGAGACATAACTTCGTATAGCATACATTATACGAACGGTAAAATGCTGGTAGCCGCAAAACT |
| Primers | Sequences (5’– 3’) |
| P8-GCW39 | AGCAAAAAGCAGGCTGACAAAT |
| PC9-GCW39 | CTCCTTCGATGTTGAGCCCTCT |
| P10-GCW39 | CTCCTTTCCAACATCCTTCGCT |
| P5-GCW42 | TACGCTGGATTTGGTAACACAC |
| P6-GCW42 | AGATCTATAACTTCGTATAATGTATGCTATACGAACGGTACTTCTGCAAAGGCCAACTGTAG |
| P7-GCW42 | GGAGACATAACTTCGTATAGCATACATTATACGAACGGTAGGATTAGTTGATGTTGCGGGAG |
| P8-GCW42 | GTTAAGCATCCGCTCAACTCTG |
| PC9-GCW42 | TCAAACCTGACCATTCGGGACA |
| P10-GCW42 | TTATTTATTCCCCCCTGATGGC |
| P5-GCW43 | TTAATAGTGCAGAGCTGTTGCTCC |
| P6-GCW43 | AGATCTATAACTTCGTATAATGTATGCTATACGAACGGTAGCCTTGTTCCCAGTATGTTTCGTC |
| P7-GCW43 | GGAGACATAACTTCGTATAGCATACATTATACGAACGGTATCTCTTCCAAATCAAATGCCGC |
| P8-GCW43 | GCACCTATGACATTTGCCAAGC |
| PC9-GCW43 | AAGGGAGATGCTTTGGGAGACT |
| P10-GCW43 | TGTTTGATAATTTGCAGTGGAG |
| P5-GCW45 | CGAACAAGACCGAACGTGGGAC |
| P6-GCW45 | AGATCTATAACTTCGTATAATGTATGCTATACGAACGGTACGGGTGACAAGTTCCAAAGAGT |
| P7-GCW45 | GGAGACATAACTTCGTATAGCATACATTATACGAACGGTATTGGGTGGATTAACCGTCGGAG |
| P8-GCW45 | AGCAGAGAATGGAGCTGTCGTA |
| PC9-GCW45 | TGGCGAACGTAAACAAAAACCT |
| P10-GCW45 | AAAAGACATTGACGAAACTGGG |
| P5-GCW46 | CTAATCTTGGTGTTTGCAGCCT |
| P6-GCW46 | AGATCTATAACTTCGTATAATGTATGCTATACGAACGGTAGGGAGACCATGCAGAAACAATT |
| P7-GCW46 | GGAGACATAACTTCGTATAGCATACATTATACGAACGGTAGGCCTTGTCGCATTCTTCATCT |
| P8-GCW46 | AAGAGAGGGTTCCAAGATCGTC |
| PC9-GCW46 | CACGTTTCCAATCTTGCCTTAG |
| P10-GCW46 | TCCAGACTTTGACCCAGGTTTC |
| P5-GCW48 | CTTCTCGCTTGCCCGATTCTAT |
| P6-GCW48 | AGATCTATAACTTCGTATAATGTATGCTATACGAACGGTAGCAAAAGTTGGCTGATGACGAC |
| P7-GCW48 | GGAGACATAACTTCGTATAGCATACATTATACGAACGGTACTCTGGTGTTTCTCTCAGTGCC |
| Primers | Sequences (5’– 3’) |
| P8-GCW48 | TGGAGACTTGGTACTTGGTGGT |
| PC9-GCW48 | TACCATACCCCGGCAGATTTCT |
| P10-GCW48 | GTACGGGGCAACAAATTATCCT |
| P5-GCW49 | GACTCGGTTCGGCCTTCTAATC |
| P6-GCW49 | AGATCTATAACTTCGTATAATGTATGCTATACGAACGGTAAAGCCCTAAAGAAAGCGAGTGC |
| P7-GCW49 | GGAGACATAACTTCGTATAGCATACATTATACGAACGGTATGGTGTTATTGGCTTGAGTGGT |
| P8-GCW49 | TCAATCTTTTGGGCAAGCACTC |
| PC9-GCW49 | TGCAATCATTGTTACCAGAGGC |
| P10-GCW49 | AGGTGGAATCGGCCAAAACTAT |
| P5-GCW51 | CGGATAAGAGATGGAGAGGCAC |
| P6-GCW51 | AGATCTATAACTTCGTATAATGTATGCTATACGAACGGTAGGCGAAGACTGGAACACTGAGC |
| P7-GCW51 | GGAGACATAACTTCGTATAGCATACATTATACGAACGGTACAGGGACGACTACTGGAAGCAC |
| P8-GCW51 | GGCATGATGGGGTCTTTTGAGG |
| PC9-GCW51 | AGTTGGATTATCAGGAGCAGCC |
| P10-GCW51 | TGTGCCCCAAAGAGTATCAATG |
| P5-GCW52 | GAAAAATAGCACCACTGGCAAG |
| P6-GCW52 | AGATCTATAACTTCGTATAATGTATGCTATACGAACGGTACCTCATTTTCTGCAAGGACCCT |
| P7-GCW52 | GGAGACATAACTTCGTATAGCATACATTATACGAACGGTATGATTTAATTACCACCCGCTGT |
| P8-GCW52 | GTTATCGTTCGATGACAGCAGG |
| PC9-GCW52 | GCAAGTCAATGCCAATGGAACA |
| P10-GCW52 | TTTGAGGCTAAAAGCGGAAGGT |
| P5-GCW53 | AAATAGTTGTGCGTGCGGTAGG |
| P6-GCW53 | AGATCTATAACTTCGTATAATGTATGCTATACGAACGGTACATGCCAATCCGTATCATCTGC |
| P7-GCW53 | GGAGACATAACTTCGTATAGCATACATTATACGAACGGTATCAGGATTGATTGGAGGGGTAG |
| P8-GCW53 | TCAATACCGGCGACTAAACTGC |
| PC9-GCW53 | CCCCTGTGAAACCCCTTATTAT |
| P10-GCW53 | ATAGGGTCACGTCGGTTTAATG |
| P5-GCW54 | TTGCTGACTCTCCCAAGACTGA |
| P6-GCW54 | AGATCTATAACTTCGTATAATGTATGCTATACGAACGGTACAACTGTAGCAGAACCGAAAGC |
| P7-GCW54 | GGAGACATAACTTCGTATAGCATACATTATACGAACGGTATCCTTCTAATGGAGCATCTTGG |
| Primers | Sequences (5’– 3’) |
| P8-GCW54 | CTGCTAAGAGCCTGACCGAGTA |
| PC9-GCW54 | TATAAGGCGAATCACAACGAAG |
| P10-GCW54 | ACTTGAAGCATCATTGTGGCAT |
| P5-GCW56 | TGTTGTCTTGCCTTTGCTCCCT |
| P6-GCW56 | AGATCTATAACTTCGTATAATGTATGCTATACGAACGGTACTCGGAAGTTGCCAAAGAAAGT |
| P7-GCW56 | GGAGACATAACTTCGTATAGCATACATTATACGAACGGTATTCGTTGCTGGTGTACTGGGAG |
| P8-GCW56 | GTCGCATCGTCACTATCGCTGT |
| PC9-GCW56 | TGTGTATTGGTCTCCCGCATCT |
| P10-GCW56 | ATTTATCTCCTCCCGTTGTTCC |
| P5-GCW58 | CACCAAAACTCTAATCAAACCG |
| P6-GCW58 | AGATCTATAACTTCGTATAATGTATGCTATACGAACGGTAGGGCGTTCTCAGTGACATCATT |
| P7-GCW58 | GGAGACATAACTTCGTATAGCATACATTATACGAACGGTATTATTGATGGGTTTGACTGCCG |
| P8-GCW58 | ATGCATTCGATGTCAAAGCTGG |
| PC9-GCW58 | AGATTCACTTGCTGGGTGCCTC |
| P10-GCW58 | AAACTGTCAGCCATAGGGGAGC |
| P5-GCW59 | CTGCCCTTGGACCTGTGTAGTT |
| P6-GCW59 | AGATCTATAACTTCGTATAATGTATGCTATACGAACGGTATGGCGATTCCGGTAATTAGTAG |
| P7-GCW59 | GGAGACATAACTTCGTATAGCATACATTATACGAACGGTAGGAGCCGACAGGAACATTTTAC |
| P8-GCW59 | TGCAGGTGATGTGCGAGGTATG |
| PC9-GCW59 | GTGGATTACCGAAACGAAAGTG |
| P10-GCW59 | TGAAGGTAGTCAAAGACCGCAG |
| P5-GCW60 | ACTTTGATGTGCTAGACTGCCT |
| P6-GCW60 | AGATCTATAACTTCGTATAATGTATGCTATACGAACGGTAACATAGAGCCAATGATGCGTTC |
| P7-GCW60 | GGAGACATAACTTCGTATAGCATACATTATACGAACGGTAGGTAGTTATTCTCGGCGTGTGT |
| P8-GCW60 | GTTGGGGTGGTGCTCTCTCTTC |
| PC9-GCW60 | TGACTGGGTTTTCGTTTATCGG |
| P10-GCW60 | AGGGCAATGCTTGTGGTTAGTT |
| P5-GCW61 | GGAGACTGGATGGTTTTGGTTG |
| P6-GCW61 | AGATCTATAACTTCGTATAATGTATGCTATACGAACGGTAAGACTTGAATTGCATTTCGGCT |
| P7-GCW61 | GGAGACATAACTTCGTATAGCATACATTATACGAACGGTACTGCCGTTCGTGCCTCTGGTAT |
| Primers | Sequences (5’– 3’) |
| P8-GCW61 | GGGCAGGAAAAACCCAATGACT |
| PC9-GCW61 | TACGGAAAATGAAATCGTGTCG |
| P10-GCW61 | ATTACGGGAGGGAATGGGAAAG |
